# Migration of a Broken Kirschner Wire after Surgical Treatment of Acromioclavicular Joint Dislocation

**DOI:** 10.1155/2016/6804670

**Published:** 2016-12-12

**Authors:** Sabri Batın, Fırat Ozan, Kaan Gürbüz, Erdal Uzun, Cemil Kayalı, Taşkın Altay

**Affiliations:** ^1^Department of Orthopedics and Traumatology, Kayseri Training and Research Hospital, Kayseri, Turkey; ^2^Department of Orthopedics and Traumatology, Izmir Bozyaka Training and Research Hospital, Izmir, Turkey

## Abstract

Kirschner wire (K-wire) is one of the commonly used implants in orthopaedics practice. Migration of the wire is one of the most frequently reported complications after fixation by the K-wire. In particular, it has been reported that a greater range of motion in the shoulder, negative intrathoracic pressure associated with respiration, gravitational force, and muscular activities may cause migration from the upper extremities. In general, thin and long foreign bodies with smooth surfaces that are localized within the tendon sheath and at an upper extremity can migrate more readily and can reach longer distances. Here, we present a patient with long-term migration of a broken K-wire who underwent fixation for acromioclavicular joint dislocation 5 years ago.

## 1. Introduction

Kirschner wire (K-wire) is one of the commonly used implants in orthopaedics practice [1]. However, there are several complications despite good outcomes with the use of K-wires [1–3]. These complications include wire loosening, tendon rupture, nerve damage, osteomyelitis, and pin tract infection [1–3]. Migration of the wire is one of the most frequent complications after fixation with a K-wire [2, 4]. Numerous fatal complications occur due to the migration of a K-wire into the mediastinum, spinal canal, heart, lungs, pulmonary artery, and aorta [1–5]. The mechanism that causes or enables K-wires to migrate is unknown, although muscle activity has been postulated as an underlying cause [4]. Here, we present a patient with long-term migration of a broken K-wire who underwent fixation for acromioclavicular (AC) joint dislocation 5 years ago.

## 2. Case Report

**Figure 1:**
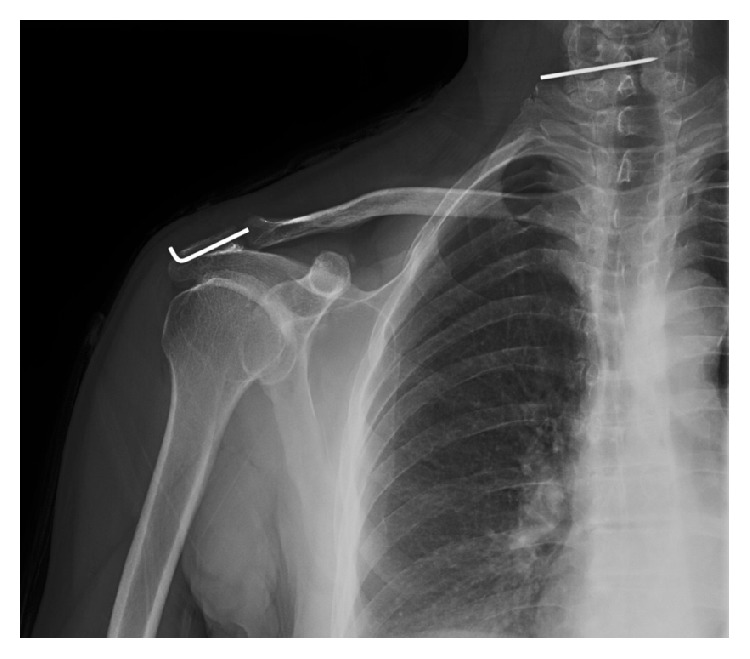
Radiographic image of migration of a broken K-wire used in the fixation of the right AC joint dislocation.

**Figure 2:**
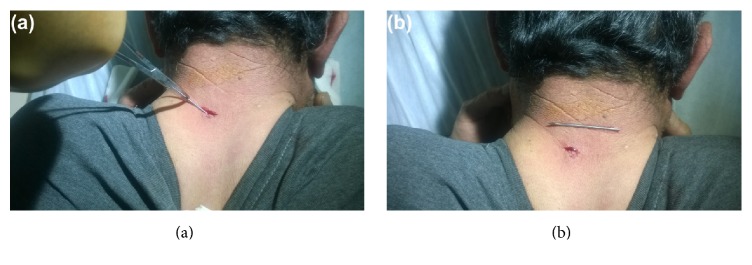
Removal of the broken tip of the K-wire from the back of the neck under local anaesthesia (a, b).

A 52-year-old man underwent surgery for an AC joint dislocation 5 years ago. The dislocation was fixed using a K-wire. The patient did not require a follow-up appointment as he had no complaints. The patient's dominant extremity was right-sided and he was a retired construction worker. The patient presented to our outpatient clinic with swelling at the back of the neck. Radiological evaluation revealed that a broken tip of the K-wire used in the fixation of the AC joint dislocation had migrated to the back of the neck (Figure 1). The broken K-wire was removed under local anaesthesia via mini-incision (Figure 2). No intervention was performed for the AC joint. The remaining broken pin was not removed from the shoulder.

## 3. Discussion

Several techniques have been described for the management of AC joint dislocations. Stabilization of the dislocated AC joint using a K-wire is one of these methods. AC joint stabilization by K-wire provides a safe and easy fixation with low morbidity; however, complications such as loss of fixation or loosening can be observed [6].

K-wire migration can result in mortality by threatening vital organs in some cases [1, 4, 7]. There are different opinions on why wires migrate. In particular, a greater range of motion in the shoulder, negative intrathoracic pressure associated with respiration, gravitational force, and muscular activities may cause migration from the upper extremities [1–4]. In general, long, thin foreign bodies with smooth surfaces that are localized within the tendon sheath and at an upper extremity can migrate more readily and can reach longer distances [8]. Such material can have a long silent period within the tissue or can cause chronic discharge, infection, or chronic pain and may damage neurovascular structures [2, 4, 8]. However, some authors have recommended that the external tips of pins or the tips of a ribbed K-wire be bent to prevent pin migration [3–5]. On the other hand, fixation with a K-wire should be performed carefully in patients >60 years with osteoporotic fracture because osteoporosis may play a role in migration [9].

Some authors emphasize that the surgical site should be monitored by radiographs at 4-week intervals and that the K-wire should be removed immediately if any sign of loosening is present [1, 3, 10]. It is often difficult to detect and remove migrated implants [2, 8]. Failure to localize migrated materials and inaccurate calculation may add difficulty to the removal procedure and lead to unnecessary tissue injury as well as prolonged operation time [2, 8].

Our patient was fortunate that no serious complication occurred after migration of the broken K-wire. It should be kept in mind that K-wire migration can be present after surgical treatment of the shoulder despite the absence of clinical complaints. Therefore, we think that periodic radiological monitoring can be helpful in preventing K-wire complications if a K-wire is used in surgery.
